# Flower senescence and other programmed cell death processes in plants: a tribute to the late Wouter G. van Doorn

**DOI:** 10.1093/jxb/erw372

**Published:** 2016-10-28

**Authors:** Sergi Munné-Bosch

**Affiliations:** Department of Evolutionary Biology, Ecology and Environmental Sciences, University of Barcelona, Spain

**Keywords:** Chromatin condensation, flower senescence, hibiscus (*Hibiscus rosa-sinensis*), petal senescence, phytoglobins, programmed cell death, rose flowers, Wouter G. van Doorn.

This collection of papers has been brought together to acknowledge the invaluable contribution of Wouter G. van Doorn (13 December 1951–16 May 2015) to plant science and the broader scientific community. Wouter was an admirable and passionate scientist who lived by the maxim ‘I have striven not to laugh at human actions, not to weep at them, nor to hate them, but to understand them’ (Baruch Spinoza). It was his PhD dissertation at the University of Utrecht entitled ‘Vascular occlusion in stems of cut rose flowers’ that opened up a whole area of experimental work aimed at understanding flower senescence and other programmed cell-death processes in plants. Wouter van Doorn’s legacy is not simply limited to his excellent series of high impact articles in basic and applied science (though these are impressive in themselves) but reaches out through the students and scientists with whom he came into contact.

**Van Doorn’s legacy**

The unique combination of interconnected knowledge generation and transfer, basic and applied science, and student training was one of the hallmarks of Van Doorn’s work. His contribution to science was based not only on establishing new concepts and findings on flower senescence and other programmed cell death processes in plants, but also on his way of understanding science and life, helping others to advance on their own.

**Figure:**
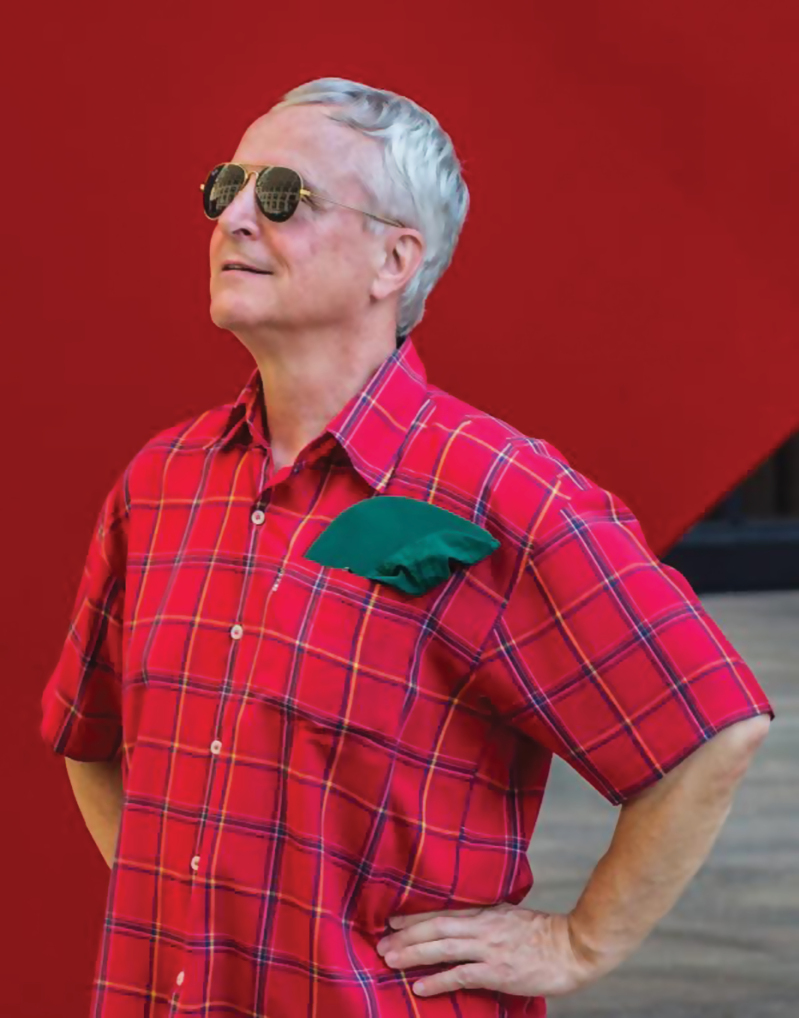

Programmed cell death (PCD) has received particular attention in plants because it is at the centre of a number of physiological processes, from germination to whole-plant senescence (especially in monocarpic plants, such as Arabidopsis), passing by other essential processes along the way, such as xylem differentiation and aerenchyma formation (Van Hautegem *et al.*, 2015). Another of these physiological processes is flower senescence, particularly the senescence of petals. Pollination usually triggers petal senescence, although the petals of some flowers also senesce in its absence. Petal senescence, as with leaf senescence (although to a lesser extent), leads to the remobilization of nutrients, which in the case of petals are transferred to the ovary for fertilization and fruit growth (Jones, 2013; Rogers and Munné-Bosch, 2016). Petal senescence is an irreversible, strictly developmentally regulated process, in which PCD plays a major role. Van Doorn and colleagues led this topic during the past decade, helping to establish a clear classification of PCD in plants (Van Doorn and Woltering, 2004, 2005, 2008, 2010; Van Doorn, 2011; Van Doorn *et al.*, 2011). In this respect, Van Doorn and Woltering (2010) argue that ‘we need not necessarily adhere to the definitions as currently in use in animal science’ and ‘to move forward in this field we need a better understanding of the genesis of autophagosome-like structures in plants … with detailed electron microscope data on the vesicles involved.’

This special issue builds on our current knowledge of PCD in plants, including reviews and new research on petal senescence. Shibuya *et al.* (2016) cover recent findings on the morphology of senescing petal cells and the regulatory mechanisms of PCD by transcription factors. Mira *et al.* (2016) discuss how phytoglobins (previously termed non-symbiotic hemoglobins, well-known nitric oxide scavengers) modulate cellular responses to auxin, cytokinin and jasmonic acid during growth and development, as well as in stress responses. Latrasse *et al.* (2016) focus their review on chromatin condensation and histone modifications associated with a major transcriptional switch occurring during PCD in response to various stimuli. Finally, Trivellini *et al.* (2016) elegantly describe the spatial and temporal dynamics of transcriptome changes during flower opening and senescence in the hibiscus flower. It is my hope that this set of papers will provide an improved understanding of petal senescence and other PCD processes in plants, helping stimulate new avenues of research in the area.

*To the memory of Wouter van Doorn: your legacy will continue in our seminars with undergraduate and graduate students*.
